# The relationship between trimethylamine-N-oxide and the risk of acute ischemic stroke: A dose‒response meta-analysis

**DOI:** 10.1371/journal.pone.0293275

**Published:** 2023-10-26

**Authors:** Yuan Hong, Zaidie Sun, Nianqiu Liu, Kai Yang, Ya Li, Qiuyue Xu, Zhangyou Guo, Yong Duan

**Affiliations:** 1 Department of Clinical Laboratory, The First Affiliated Hospital of Kunming Medical University, Yunnan Key Laboratory of Laboratory Medicine, Yunnan Province Clinical Research Center for Laboratory Medicine, Kunming, People’s Republic of China; 2 Department of Breast Surgery, The Third Affiliated Hospital of Kunming Medical University, Yunnan Cancer Center, Kunming, Yunnan, People’s Republic of China; 3 Department of Minimally Invasive Interventional Medicine, The Third Affiliated Hospital of Kunming Medical University, Yunnan Cancer Center, Kunming, Yunnan, People’s Republic of China; Satyawati College, University of Delhi, INDIA

## Abstract

**Background:**

Although trimethylamine-N-oxide (TMAO) shows a notable correlation with cardiovascular disease, its association with acute ischemic stroke (AIS) remains uncertain and necessitates further investigation.

**Objective:**

A meta-analysis was conducted to assess the relationship between trimethylamine-N-oxide and acute ischemic stroke.

**Methods:**

We conducted a comprehensive search in PubMed, Embase, Cochrane, CNKI, VIP, Wanfang, and CBM, spanning from their inception to 23 September 2023. The search was consistently updated and supplemented by bibliographies of retrieved articles and previous reviews. A total of 20 eligible studies, including 17 case‒controls and 3 cohort studies, were selected, involving 9141 participants (5283 case group, 3858 control group). For the dose‒response analysis, three case–control studies were eligible. We extracted and pooled TMAO mean and standard deviation from observational studies for control and ischemic stroke groups. The effect sizes were combined using the random-effects model. Where possible, dose‒response analysis was performed.

**Result:**

Overall, the pooled standardized mean difference (SMD) demonstrated significantly higher concentrations of serum/plasma TMAO in AIS compared to the control group (SMD = 1.27; 95% CI: 0.9, 1.61, P<0.001). Additionally, the dose‒response meta-analysis revealed a 12.1% relative increase in the risk of acute ischemic stroke per 1 μmol/L rise in TMAO concentration (RR = 1.12; 95% CI 1.07–1.17; P<0.05; I^2^ = 1.6%, P = 0.4484).

**Conclusion:**

These findings indicate a potential increased risk of AIS associated with elevated TMAO levels.

## 1. Introduction

Stroke is a significant global public health issue, being a major contributor to both mortality and disability [1, 2]. Clinically, stroke is defined as a group of diseases resulting in permanent neuronal damage or death due to a lack of blood supply to a specific region of the brain. It can be categorized into two main types: ischemic stroke (IS) and hemorrhagic stroke [3]. Among these, IS is the most common subtype, accounting for approximately 85% of all strokes. It primarily results from vascular blockage, leading to ischemic damage to brain tissue, which includes both cerebral thrombosis and cerebral embolism. The Trial of Org 10172 in Acute Stroke Treatment (TOAST) classification further categorizes IS into several subtypes, such as large-artery atherosclerosis, small-vessel occlusion, cardioembolic, other determined cause, or undetermined cause [4].

Gut microbes metabolize choline-rich foods to generate trimethylamine (TMA), subsequently converted to trimethylamine-N-oxide (TMAO) in the liver by flavin monooxygenase family members, FMO1 and FMO3 [5, 6]. Indeed, research has demonstrated that Trimethylamine N-Oxide (TMAO) contributes to the development of atherosclerosis through several mechanisms, including enhanced Macrophage Cholesterol Accumulation and Foam Cell Formation, Pro-Inflammatory Changes in the Arterial Wall, Increased Platelet Hyperresponsiveness and Increased Potential for Arterial Thrombosis [7–9]. Currently, some studies have highlighted the association between TMAO and IS. For instance, research has shown that an increase in peripheral blood TMAO concentration is dose-dependently related to an elevated risk of first-time IS [10]. Another nested case-control study, after adjusting for choline, L-carnitine, and baseline systolic blood pressure, found that higher levels of TMAO in hypertensive individuals were associated with an increased risk of IS [11]. However, there are also controversial findings. For example, in comparison to the asymptomatic group, patients with IS or transient ischemic attacks showed not an increase but a decrease in peripheral blood TMAO concentration [12]. Furthermore, the results of another study indicated that the plasma TMAO levels in atherosclerotic ischemic stroke patients were significantly lower than those in the healthy control group [13]. To our knowledge, previous meta-analysis studies have primarily focused on composite cardiovascular endpoints, including myocardial infarction, heart failure, stroke, and mortality. There is currently no comprehensive investigation into the relationship between TMAO and AIS risk, especially in terms of dose-response. Therefore, this study aims to provide more evidence-based medicine by conducting a systematic review of previously published literature and using meta-analysis techniques to explore the relationship between peripheral blood TMAO levels and AIS, with a particular focus on the dose-response relationship between the two.

## 2. Materials and methods

### 2.1 Search strategy

We conducted a comprehensive literature search in PubMed, Embase, VIP, CNKI, CBM, Wanfang Database, and Cochrane Library, covering the period from inception to 23 September 2023. The search strategy employed the following terms: (1) Trimethyloxamine, including Medical Subject Headings (MeSH) terms trimethylammonium oxide, trimethylamine N-oxide, TMAO, and trimethylamine oxide; (2) Ischemic Stroke, encompassing MeSH terms ischemic strokes, Stroke, Ischemic, Ischaemic Stroke, Ischaemic Strokes, Stroke, Ischaemic, Cryptogenic Ischemic Stroke, Cryptogenic Ischemic Strokes, Ischemic Stroke, Cryptogenic, Stroke, Cryptogenic Ischemic, Cryptogenic Stroke, Cryptogenic Strokes, Stroke, Cryptogenic, Cryptogenic Embolism Stroke, Cryptogenic Embolism Strokes, Embolism Stroke, Cryptogenic, Stroke, Cryptogenic, Embolism, Wake-up Stroke, Stroke, Wake-up, Wake up Stroke, Wake-up Strokes, Acute Ischemic Stroke, Acute Ischemic Strokes, Ischemic Stroke, Acute, Stroke, and Acute Ischemic; and (3) Stroke, including MeSH terms Strokes, Cerebrovascular Accident, Cerebrovascular Accidents, CVA (Cerebrovascular Accident), CVAs, Cerebrovascular Accident, Cerebrovascular Apoplexy, Apoplexy, Cerebrovascular, Vascular Accident, Brain, Brain Vascular Accident, Brain Vascular Accidents, Vascular Accidents, Brain, Cerebrovascular Stroke, Cerebrovascular Strokes, Stroke, Cerebrovascular, Strokes, Cerebrovascular, Apoplexy, Cerebral Stroke, Cerebral Strokes, Stroke, Cerebral, Strokes, Cerebral, Stroke, Acute, Acute Stroke, Acute Strokes, Strokes, Acute, Cerebrovascular Accident, Acute, Acute Cerebrovascular Accident, Acute Cerebrovascular Accidents, Cerebrovascular Accidents, and Acute. These terms were combined as follows: 1 and (2 or 3). The search history is available in the S1 Table. Furthermore, we reviewed the reference lists of retrieved articles to identify additional relevant studies. Two independent authors (Z.S. and Y.H.) screened the titles, abstracts, and full texts of all identified articles. Any disagreements were resolved by a third investigator (Z.G.).

### 2.2 Selection criteria

The inclusion criteria for the meta-analysis were as follows: (1) Study participants were adults aged ≥ 18. (2) The exposure factor was TMAO, and studies reported either the mean and standard deviation of TMAO for the two groups or the odds ratio with corresponding 95% confidence interval for different increments of TMAO. (3) The included studies focused on ischemic stroke patients, adhering to internationally accepted guidelines for defining and describing acute ischemic stroke. (4) The selected articles were observational studies, including cross-sectional, case‒control, and cohort studies. Exclusion criteria were as follows: (1) Retrieved articles included reviews, review articles, conference papers, letters to editors, case reports, meta-analyses, animal experiments, or unrelated studies. (2) Articles lacking mean and standard deviation data for TMAO levels in the case and control groups or relevant data to calculate them. (3) Studies that aggregated outcomes into total stroke without reporting AIS separately. (4) Insufficient availability of detailed study information.

Two researchers independently evaluated the eligibility of the literature. In case of any disagreements, a third researcher was consulted, or a group discussion was held to reach a consensus. The protocol for this review was registered with PROSPERO (CRD42022373779).

### 2.3 Data extraction

Following the PRISMA guidelines, two authors (Z.S. and Y.H.) extracted the following characteristics from the included studies: first author’s surname, publication time, study origin, cohort name, sample size, number of cases, age at entry, sex distribution, outcome, method of measuring TMAO, and the mean and standard deviation of TMAO for both control and AIS patients separately. All specimens were collected promptly after admission. For the dose‒response meta-analysis, risk estimates (preferably adjusted measures), hazard ratios (HRs), risk ratios (RRs), or odds ratios (ORs) with their corresponding 95% confidence intervals (CIs) were utilized, along with adjustment factors. In cases where multiple risk estimates were provided, the multivariable model was chosen. Study quality assessment was conducted using the Newcastle–Ottawa Quality Assessment Scale (NOS), which evaluates three quality parameters (selection, comparability, and outcome) divided into eight specific items. Studies scoring ≤ 3 were classified as low quality, 4–6 as moderate quality, and ≥ 7 as high quality [14].

### 2.4 Strategy of data synthesis

The statistical analyses were conducted using STATA V.14 (Stata Corp LP, College Station, TX, USA). The primary studies reported TMAO concentration in various formats, either as a continuous or categorical variable. To ensure consistency across the meta-analysis, we extracted the mean and standard deviation of TMAO for the control and AIS groups from 19 selected articles. In cases where this data was not available, we applied the transformation method proposed by Dehui Luo et al. [15, 16]. Heterogeneity was assessed using the I^2^ value, with values below 25% indicating low heterogeneity, 25–50% indicating moderate heterogeneity, and above 50% indicating high heterogeneity [17]. The results were combined using the random-effects model in the presence of significant heterogeneity; otherwise, the fixed-effects model was employed. For the dose‒response meta-analysis between TMAO and the risk of AIS, we utilized the generalized least-squares trend estimation method proposed by Orsini et al. [18]. This method required the OR (with 95% CI), the median/mean level of exposure, and the number of cases and total participants from each study. In cases where the median or mean TMAO doses were not provided, we estimated the midpoint of each category. If a category was open-ended, we calculated the midpoint by assuming it had the same interval as the adjacent category [19]. To explore potential nonlinear associations between TMAO levels and AIS risk, we employed restricted cubic splines with three knots.

A meta-regression analysis was employed to investigate how significant covariates might impact the heterogeneity observed between studies. We established statistical significance by considering P values equal to or less than 0.05 [20]. Subgroup analyses were carried out to investigate plausible origins of heterogeneity, taking into account variables such as study type, publication year, sample size, and the methodology for detecting TMAO. When no significant covariates were identified as contributors to heterogeneity, sensitivity analyses were performed to gauge the impact of each individual study on the overall effect size. Each study was methodically excluded one at a time to evaluate its potential influence. To ascertain the presence of publication bias, we utilized funnel plots, Egger’s test, and Begg’s test. If publication bias was detected, we addressed its effects using a clipping method.

## 3. Result

### 3.1 Literature search and characteristics of included studies

**Fig 1:**
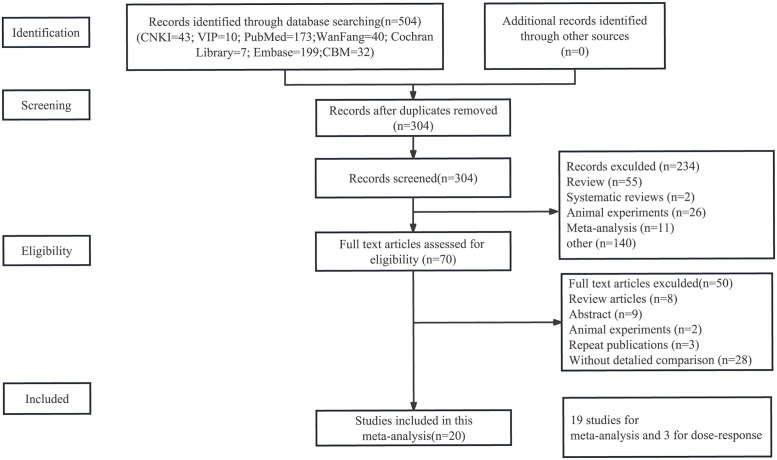
Literature search and study selection process for inclusion in meta-analysis of trimethylamine-N-oxide (TMAO) and the risk of acute ischemic stroke (AIS).

A comprehensive computerized database search initially identified a total of 504 papers, with the search and screening process illustrated in Fig 1. After evaluating the full-text readings of 70 articles, a final selection of 20 articles [10, 11, 13, 21–37] met the inclusion criteria for the meta-analysis. Among these, 17 were case‒control studies and 3 were cohort studies, involving a total of 9,141 participants. Nineteen studies were conducted in China, while one study was conducted in the United States. TMAO determination methods included HPLC‒MS, LC‒MS, and ELISA. Nineteen articles were included in the meta-analysis, and three articles were included in the dose‒response analysis. Tables 1 and 2 provide the essential characteristics of the included studies. The level of agreement between the two investigators in data collection, as measured by the Kappa statistic, was 0.619.

**Table 1:** Basic features of the involved literature for meta-analysis.

Authors and years	Country	Type of study	Sample (AIS/control)	Sample type	Method*	Sex (male/female)		Age (years)		Means of AIS group	SDs of AIS group	Means of Control	SDs of Controls	Scores of NOS
						AIS	control	AIS	control					
Xiaohui Zhao 2021	China	Case‒controls	124/118	Plasma	HPLC‐MS/MS	/	/	56.9±3.2	57.4±3.5	13.97	1.26	7.95	3.18	6
Yunyun Xu 2022	China	Case‒controls	108/59	Plasma	LC‒MS	66/42	23/36	66.42±3.16	62.57±1.68	2763	408.7	1642	144.1	6
Yanfei Xu 2017	China	Case‒controls	60/30	Plasma	HPLC‐MS/MS	45/15	023/7	59(19.75)	57(15)	202.09	144.21	140.88	48.12	7
Kaicheng Wang 2020	China	Case‒controls	108/59	Plasma	LC‒MS	/	/	66.42±3.16	62.57±1.68	3205.51	716.39	1663	117.8	6
Jiaxin Luo 2022	China	Case‒controls	137/121	Plasma	ELISA	72/65	35/86	57.9±8.0	55.4±10.00	3.21	0.33	2.86	0.31	7
Zhendong Liu 2017	China	Case‒controls	80/40	Plasma	LC‒MS	48/32	27/13	62.5±8.4	61.4±8.6	14.86	4.11	6.12	3.84	6
Shuoxi Liao 2016	China	Case‒controls	322/231	Plasma	LC‒MS	220/102	130/101	61(19)	56(11)	2.7	2.57	1.9	1.47	6
Qijin Zhai 2020	China	Case‒controls	408/102	Serum	LC‒MS	233/175	55/47	67.9±9.1	66.3±8.8	3.6949	1.8599	3.0056	1.7296	6
Chen Zhu 2019	China	Case‒controls	256/100	Plasma	LC‒MS	139/117	/	67.1±11.0	/	5.6	2.4	4.9	1.8	8
Jianli Zhang 2021	China	Cohort study	351/150	Plasma	LC/MS	177/174	75/75	66(57–74)	66(57–74)	6.5908	4.6152	4.1054	2.6199	6
Dongjuan Xu 2021	China	Case‒controls	50/50	Plasma	LC‒MS	35/15	14/36	63.2±12.0	57.36±10.65	2659.51	976.81	1484.75	648.87	7
Chuanjie Wu 2020	China	Case‒controls	377/50	Plasma	LC‒MS	215/162	/	62.5±10.7	/	5.3454	3.2	3.2477	1.7556	8
Taoping Sun 2021	China	Case‒controls	953/953	Plasma	HPLC‐MS/MS	544/409	544/409	63.14(9.26)	63.32(8.47)	3.0706	2.1159	2.5751	1.7076	8
Schneider C 2020 C. Schneider 2020	USA	Cohort study	193/100	Plasma	LC‒MS	122/71	53/47	69(60–78)	65(57–75)	4.5042	2.7039	3.4838	2.3168	7
Maimaiti Rexidamu 2019	China	Case‒controls	255/255	Serum	HPLC‒MS/MS	136/119	/	65(57–71)	/	6.3963	4.9952	4.3209	2.8331	8
Zhaoguang Liang 2019	China	Case‒controls	68/111	Plasma	HPLC‐MS/MS	40/28	64/47	68.0±9.6	64.1±13.3	8.25	1.58	2.22	0.09	7
Zaiwang Li 2022	China	Case‒controls	108/60	Plasma	HPLC‐MS/MS	/	/	/	/	3.6527	3.1934	1.9007	1.4888	7
Yan-Yan Chen 2022	China	Case‒controls	291/235	Plasma	LC‒MS	132/159	98/137	61.68±7.27	59.71±7.67	129.65	46.24	85.15	32.11	7
Dong Liu 2023	China	Cohort study	412/412	Plasma	HPLC‐MS/MS	189/223	189/223	69.3(62.7,75.3)	69.3(62.9,75.2)	3.6669	2.3888	3.0152	1.6515	7
Jing Nie 2018	China	Case‒controls	622/622	Serum	LC‒MS	292/330	292/330	62.2(7.3)	62.2(7.3)	/	/	/	/	8

* HPLC‐MS/MS is high performance liquid chromatography-mass spectrometry or mass spectrometry. LC-MS is liquid chromatography-mass spectrometry, and ELISA means enzyme linked immunosorbent assay. AIS, acute ischemic stroke; SDs, Standard deviations; NOS, New Ottawa Scale.

**Table 2:** Basic features of the involved literature for dose‒response meta-analysis.

Study	Country	Study type	Sample (AIS/Control)	Sex (male/female)	TMAO comparison (μmol/L)	OR (95% CI)	Adjusted variables
Taoping Sun 2021	China	Case‒control	953/953	1088/818	Quintile 4 vs. 1 (<1.53 v >3.83)	1.81 (1.27, 2.59)	Adjusted for age, sex, BMI, smoking status, alcohol habit, history of hypertension, history of diabetes, triglycerides, LDL-cholesterol, and HDL-cholesterol.
Dong Liu 2023	China	Case‒control	412/412	378/446	Quintile 4 vs. 1 (<1.97 v >4.19)	1.74 (1.16, 2.61)	Adjusted for body mass index (continuous), smoking (yes or no), hypertension (yes or no), educational attainment (0 year, 1–5 years, or ≥6 years), and estimated glomerular filtration rate (quartiles).
Jing Nie 2018	China	Case‒control	502/506	NA.	Quintile 3 vs. 1 (<1.79 v ≥1.79)	1.35 (1.00, 1.81)	Adjusted for SBP, BMI, fasting glucose, total cholesterol, eGFR, total homocysteine, folate, smoking status at baseline, time-averaged SBP during the treatment period, choline and L-carnitine

AIS, acute ischemic stroke; BMI, body mass index; CI, confidence interval; eGFR, estimated glomerular filtration rate; HDL, high-density lipoprotein; LDL, low- density lipoprotein; NA, not available; OR, odds ratio; SBP, systolic blood pressure; TMAO, trimethylamine-N-oxide.

All of the included studies obtained a rating of 6 to 8 stars on the Newcastle‒Ottawa Scale. Specifically, six studies received a score of 6, eight studies received a score of 7, and five studies received a score of 8. The agreement between the two investigators for quality assessment, as measured by the Kappa statistic, was 0.922.

### 3.2 Meta-analysis of TMAO and AIS risk

**Fig 2:**
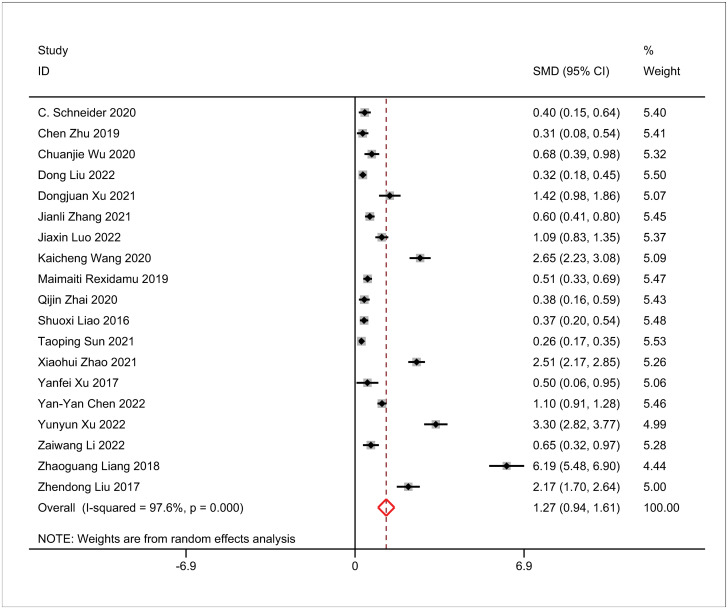
Impact of trimethylamine-N-oxide (TMAO) plasma levels and acute ischemic stroke (AIS) risk.

In this meta-analysis, a considerable degree of heterogeneity was observed among the included studies (I^2^ = 97.6%, P<0.01), indicating a notable difference in effect size between the AIS group and the control group using a random effects model. The analysis revealed a higher effect size in the AIS group compared to the control group (SMD = 1.27, 95% CI 0.94, 1.61, P<0.01; see Fig 2).

**Fig 3:**
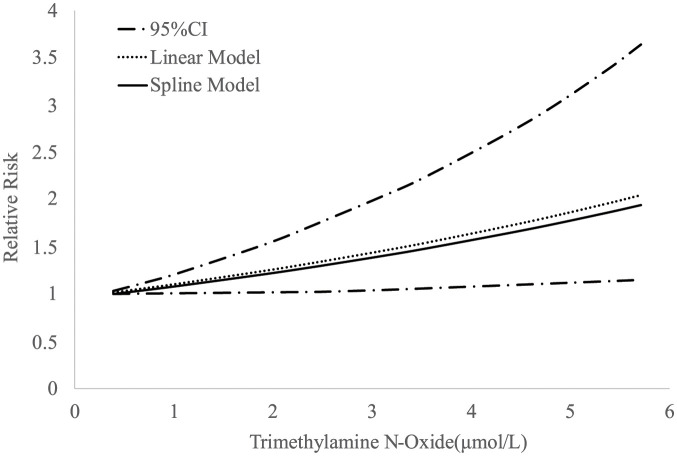
Dose–response associations between (trimethylamine-N-oxide) TMAO and AIS risk.

The dose‒response meta-analysis revealed a noteworthy correlation between TMAO levels and AIS risk, showing a 12.0% increase in relative risk (RR) for AIS with each 1 μmol/L increment of TMAO (RR = 1.12, 95% CI 1.07, 1.17, P<0.01, I^2^ = 1.60%, P = 0.4484; based on three studies; see Fig 3).

### 3.3 Subgroup analysis and meta-regression analysis

**Fig 4:**
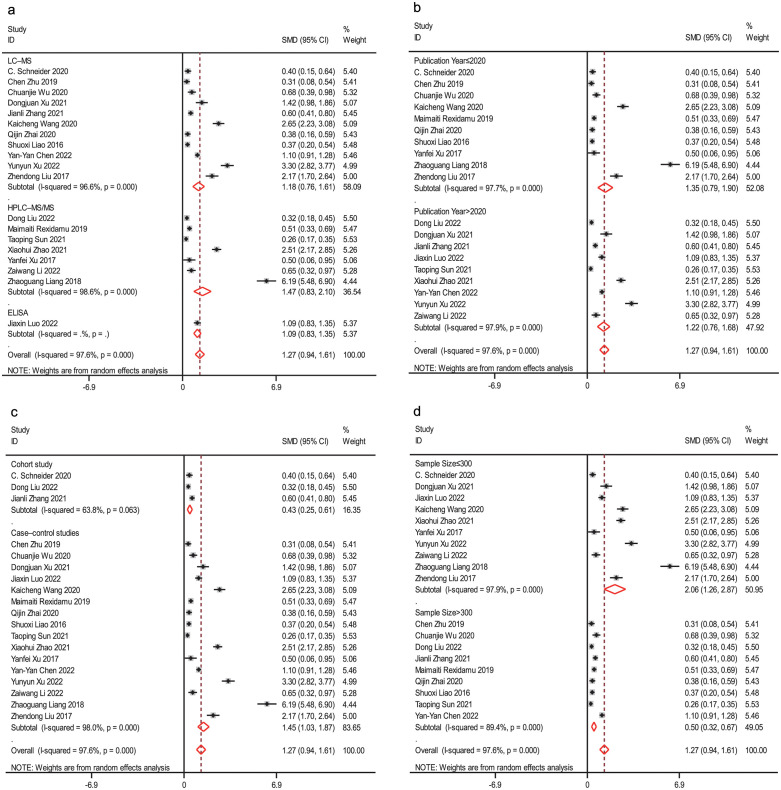
Subgroup analyses of the impact of trimethylamine-N-oxide (TMAO) plasma levels on the risk of AIS. Random effects standard mean difference (SMD) and 95% confidence interval (CI) for the risk of acute ischemic stroke (AIS) in HPLC‒MS/LC‒MS/ELISA (a), SMD and 95% CI for the risk of AIS in publication years >2020 and ≤2020 (b), SMD and 95% CI for the risk of AIS in different types of studies (c), and SMD and 95% CI for the risk of AIS in different sample sizes (d).

Subgroup analysis was conducted to account for the high heterogeneity of the included literature. The analysis focused on study type, publication period, TMAO detection method, and total sample size, while univariate regression analysis considered the basic characteristics of the included literature (refer to Fig 4 and Table 3). Among the included studies, one was conducted in the USA, while the remaining studies were conducted in China. Removing this article did not significantly affect the heterogeneity, indicating that the region was not a source of heterogeneity. In the subgroup analysis of the TMAO assay, the total effect standardized mean difference (SMD) showed substantial heterogeneity (HPLC‒MS: I^2^ = 98.6%, P<0.001; LC‒MS: I^2^ = 96.5%, P<0.001; see Fig 4a), with only a slight change in effect size (HPLC‒MS: SMD = 1.72, 95% CI 1.07, 2.38; LC‒MS: SMD = 1.10, 95% CI 0.68, 1.51; ELISA: SMD = 0.38, 95% CI 0.16, 0.59). When analyzing publication years, high heterogeneity was observed before and after 2020 (>2020: I^2^ = 97.9%, P<0.001; LC‒MS: I^2^ = 97.7%, P<0.001; see Fig 4b), with no significant change in effect size (>2020: SMD = 1.22, 95% CI 0.76, 1.68; ≤2020: SMD = 1.35, 95% CI 0.79, 1.90). Heterogeneity was high in the case‒control subgroup (I^2^ = 97.6%, P<0.001), while it was lower in the cohort study subgroup (I^2^ = 63.8%, P = 0.63). The effect size appeared to be lower in the cohort study subgroup (case‒control: SMD = 1.45, 95% CI 1.03, 1.87; cohort study: SMD = 0.43, 95% CI 0.25, 0.61; see Fig 4c), but both groups exhibited higher heterogeneity. Furthermore, subgroup analysis based on sample size showed no significant heterogeneity (≤300: I^2^ = 97.9%, P<0.001; >300: I^2^ = 89.4%, P<0.001; see Fig 4d), with a notable difference in effect size (≤300: SMD = 2.06, 95% CI 1.26, 2.87; >300: SMD = 0.50, 95% CI 0.32, 0.67). However, the subgroup analyses did not identify any specific sources of heterogeneity.

**Table 3:** Meta-regression analysis for the risk of acute ischemic stroke (AIS).

	P value	Number of studies included in the analysis
Region	0.533	19
Type of study	0.268	19
Detection method	0.788	19
Publication year	0.687	19
Total sample size	0.109	19
Male (%) for AIS	0.755	17
Male (%) for control	0.972	13
Mean age for AIS	0.708	18
Mean age for control	0.983	15

Furthermore, a meta-regression analysis was conducted to investigate the influence of baseline characteristics on the relationship between TMAO and AIS risk. No significant effects were observed in the analysis (all P values > 0.1), consistent with the results obtained from the subgroup analysis.

### 3.4 Sensitivity analyses

**Fig 5:**
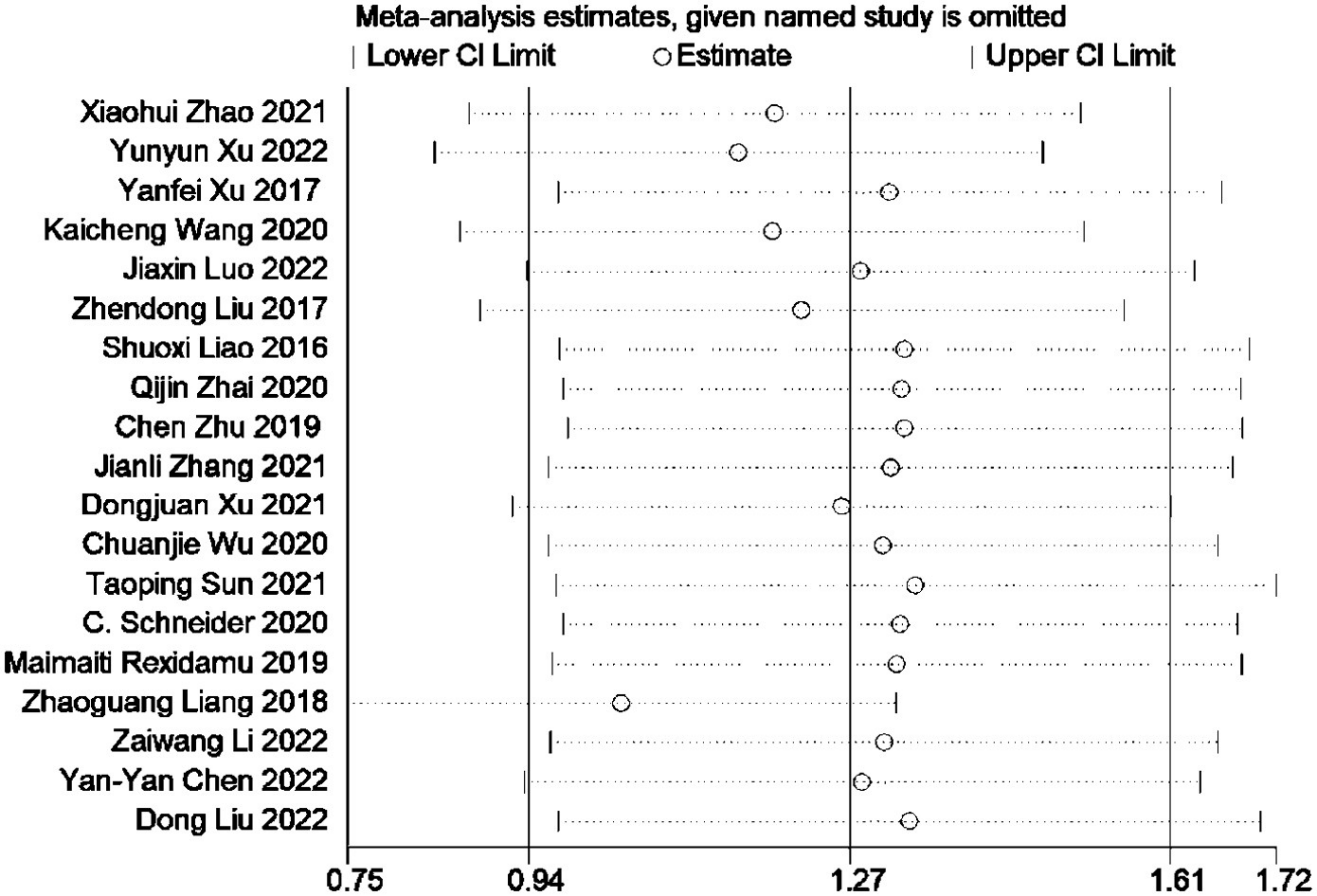
Sensitivity analysis was conducted by removing each study in turn and recalculating the pooled standard mean difference estimates.

Sensitivity analysis was performed by comparing the combined effect size (SMD, 95% CI) to assess the robustness of the results. The analysis revealed that removing any single study did not overturn the findings, indicating the reliability of the combined results (see Fig 5).

### 3.5 Analysis of publication bias

**Fig 6:**
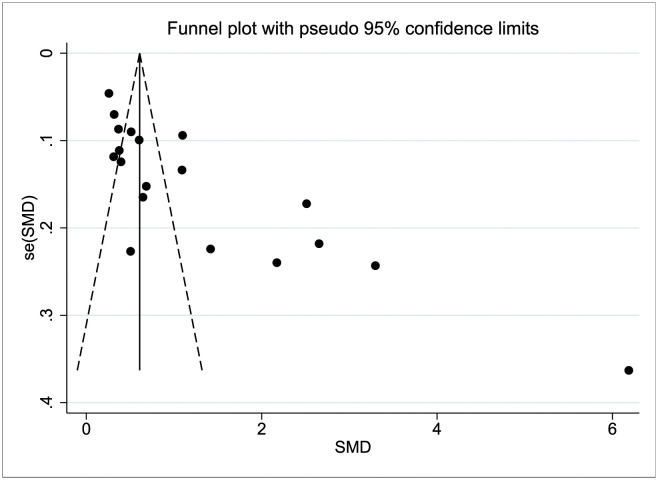
Correlation of trimethylamine-N-oxide (TMAO) and risk of acute ischemic stroke (AIS) development meta-analysis publication bias funnel plot.

The funnel plot exhibited asymmetry, suggesting the presence of publication bias in the included studies (see Fig 6). To evaluate publication bias, Begg’s and Egger’s tests were conducted, both indicating significant publication bias (all P<0.001). To assess the potential impact of publication bias on the overall effect size, a sensitivity analysis was performed by incorporating nine hypothetical studies using the cut-and-mend method. The re-estimated effect size did not exhibit a significant change, and the difference was not statistically significant (SMD = 1.47, 95% CI 1.02, 2.12, P = 0.946). These findings suggest that the presence of publication bias did not exert a substantial influence on the overall effect size and the robustness of the analysis.

## 4. Discussion

This study represents the first comprehensive systematic review and dose‒response meta-analysis investigating the relationship between circulating levels of trimethylamine N-oxide (TMAO) and the risk of acute ischemic stroke (AIS), based on a thorough search of meta-analyses listed on the PROSPERO platform. A total of 19 relevant articles were meticulously selected for inclusion in this analysis. The findings indicate a significant elevation in serum/plasma TMAO levels among AIS patients compared to the control group, suggesting the potential involvement of TMAO in the development of AIS. Furthermore, the dose‒response meta-analysis, incorporating three studies, demonstrated a consistent 12.1% relative increase in AIS risk for each additional 1 mol/L of TMAO, indicating a linear correlation between TMAO levels and the risk of AIS. Importantly, this strong association between TMAO plasma levels and AIS risk remained consistent across all tested subgroups and study populations, underscoring the robustness and generalizability of the findings. These results highlight the potential of TMAO as a valuable biomarker for identifying individuals at high risk of AIS and suggest it may also serve as a promising therapeutic target for reducing the risk of AIS.

Randomized controlled trials (RCTs) are widely regarded as the gold standard for establishing causal relationships between interventions and outcomes. However, due to the limitations of available evidence, our study relied on observational studies, including case‒control and cohort studies, which are susceptible to selection bias, recall bias, and reverse causality. It is worth noting that the cohort subgroup consisted of only three articles, potentially introducing bias. The study design itself can significantly influence the correlation between TMAO levels and AIS risk. Although our meta-analysis provides evidence of an association between circulating TMAO levels and AIS risk, it is important to interpret these findings cautiously given the inherent limitations of the included studies. Regarding heterogeneity, the cohort study exhibited high heterogeneity exceeding 50%. However, the heterogeneity decreased significantly, suggesting that the study design may contribute to observed heterogeneity to some extent. Nonetheless, due to the limited number of articles included, further confirmation is required. Furthermore, sensitivity analyses indicated that the results were robust to the removal of specific regions. However, it is worth noting that only one of the included articles was conducted in the United States, where dietary patterns and sources of TMAO may differ from those in China and other regions. TMAO is primarily obtained from foods containing choline or carnitine, and variations in dietary structures may result in divergent TMAO levels. However, due to the limited number of studies in our analysis, we were unable to thoroughly explore whether the correlation between TMAO and dietary patterns would vary across regions. Notably, high-quality investigations examining the association between TMAO and cardiovascular risk have primarily been conducted in the United States and Europe. Despite potential differences in dietary patterns and lifestyle factors, these studies consistently demonstrate a positive correlation between TMAO levels and the risk of cardiovascular disease. Therefore, additional investigations encompassing diverse regions and populations are crucial to verify and strengthen our current understanding of this relationship. Although subgroup analyses and one-way regression analyses were performed to explore potential influencing factors, neither method revealed any significant factors affecting the findings. Moreover, the dose‒response analysis adjusted for various AIS-related risk factors, including blood pressure, renal function, and BMI, and the results still indicated a positive association between TMAO levels and AIS prevalence. It is important to acknowledge that publication bias was detected in our analysis. However, the cut-and-patch method was employed to verify that the bias had minimal impact on the results. Additionally, our study included both published and unpublished studies to mitigate the influence of publication bias. It is possible that negative results tend to remain unpublished, which could explain the inclusion of unpublished literature in our study.

Ischemic stroke can arise from various events, such as cardiac embolism, cerebral vascular occlusion, and atherosclerosis, all affecting cerebral circulation [38]. Previous meta-analyses have demonstrated a positive dose-dependent relationship between plasma levels of trimethylamine N-oxide (TMAO) and the risk and mortality of cardiovascular events, including stroke [19]. In a meta-analysis encompassing 23 observational studies investigating the connection between TMAO and stroke, increased TMAO levels were associated with a 68% higher risk of stroke (OR 1.83; 95% CI 1.02–3.29; P = 0.04). Additionally, the mean TMAO concentrations in stroke patients were found to be 2.20 μmol/L higher than in non-stroke controls (MD 2.20; 95% CI 1.23–3.16; P < 0.00001) [39]. Although the precise mechanism linking elevated TMAO levels to the increased risk of ischemic stroke has not been fully elucidated, multiple studies have established a close association between TMAO and the development of atherosclerosis. This association manifests through various mechanisms, including the inhibition of bile acid synthesis [40], platelet activation and its potential role in thrombosis [7], activation of pro-inflammatory pathways [41], etc. TMAO may promote thrombosis by enhancing tissue factor expression and activity, as well as increasing thrombin production [42]. Conversely, it can also reduce the formation of atherosclerotic thrombi by inhibiting TMAO production through Ca2+ regulation [43, 44]. Moreover, TMAO directly enhances platelet response to different agonists (ADP, thrombin, and collagen) and accelerates thrombosis [7]. Another plausible explanation is that TMAO induces macrophage activation and promotes foam cell formation, which is associated with atherosclerotic plaque instability [45]. Hypertension is a significant risk factor for acute ischemic stroke. Other risk factors include a history of transient ischemic attacks (TIAs), smoking, high cholesterol, diabetes, obesity, end-stage kidney disease, and atrial fibrillation [46]. Numerous studies have highlighted the crucial link between TMAO and hypertension [47, 48]. A systematic review and meta-analysis have established a significant positive dose-dependent correlation between circulating TMAO concentration and the prevalence of hypertension. The risk ratio (RR) for hypertension prevalence increases by 9% per 5-μmol/L increment and 20% per 10-μmol/L increment of circulating TMAO concentration [49]. These findings partly explain the elevation in plasma TMAO levels in patients with ischemic stroke compared to healthy controls. However, the association between circulating levels of TMAO and ischemic stroke remains a topic of debate. For example, a case‒control study focusing on individuals with atherosclerotic stroke of the aortic arteries reported notably lower TMAO concentrations in patients with ischemic stroke or transient ischemic attack compared to the asymptomatic group. Nonetheless, caution is necessary when interpreting these findings as preexisting stroke or treatment may have influenced TMAO concentrations, and the selection of patients and controls was not well-balanced [12].

It is crucial to acknowledge the limitations of this study. The majority of included studies were case‒control studies, with a limited number of cohort studies and no randomized controlled trials. Further research, particularly RCTs, will be necessary to establish a causal relationship between TMAO and AIS risk. Additionally, future studies should strive to minimize potential sources of bias. While the included studies received good quality assessments according to the Newcastle‒Ottawa Scale, the reliance on predominantly observational designs may impact the reliability of the meta-analysis results. Moreover, there was considerable heterogeneity in the included literature, and subgroup and regression analyses were conducted to explore the potential sources of heterogeneity. However, it is important to validate these findings further, as heterogeneity could be influenced by variations in study types. The evaluation of the final level of evidence for the results was not performed, and existing research on trimethylamine N-oxide (TMAO) suggests the presence of sex, age, and ethnic differences. The patients and healthy controls included in this study were predominantly older individuals, while one-third of ischemic strokes occur in young and middle-aged populations. This disparity may introduce changes in the results. Additionally, the majority of the included studies were conducted in China, and the differences between Chinese and Western diets could potentially affect TMAO levels and their association with ischemic stroke. Furthermore, TMAO lacks a standardized reference range or detection method, which may limit the generalizability of the results. Future research should aim to address these limitations by including a more diverse range of study designs, considering different populations and age groups, and standardizing TMAO measurement techniques and reference ranges.

Our study offers several notable advantages. Firstly, it stands as the pioneering analysis to incorporate a dose‒response assessment of the association between TMAO and AIS. This unique approach allows for a comprehensive understanding of the relationship between TMAO levels and the risk of AIS, providing valuable theoretical groundwork for future investigations. Secondly, the exclusion of overlapping studies assures that they did not exert a substantial influence on the final results, thereby reinforcing the robustness and reliability of our findings.

## 5. Conclusion

Our investigation unveiled a compelling association between elevated TMAO levels and the risk of AIS. The dose‒response analysis indicated a significant 12.1% increase in AIS risk for each 1 mol/L increment in peripheral blood TMAO levels. However, it is crucial to exercise caution when generalizing these results due to the considerable heterogeneity and potential biases inherent in the literature. To establish a more comprehensive understanding, further validation through improved research design, larger sample sizes, and inclusion of diverse age groups is warranted. Additionally, meticulous control for confounding factors is essential to elucidate the potential causal relationship between TMAO and AIS.

## Supporting information

S1 Table Key terms for search of electronic databases. (PDF)

S2 Table PRISMA 2020 checklist. (PDF)

S3 Table The relevant data extracted. (PDF)
