# Supplementary material for: The relationship between trimethylamine-N-oxide and the risk of acute ischemic stroke: A dose‒response meta-analysis
Source: PLoS One. 2023 Oct 26;18(10):e0293275. doi: 10.1371/journal.pone.0293275 (PMC10602245; doi:10.1371/journal.pone.0293275)
Supplement: S1 Table — (PDF) [file pone.0293275.s001.pdf]

Table S1. Key terms for search of electronic databases.	
Key terms for search of PubMed	
#1	(("trimethyloxamine" [Supplementary Concept]) OR ((((trimethylammonium oxide) OR (trimethylamine N-oxide)) OR (trimethylamine N-oxide)) OR (TMAO))) AND ((("Ischemic Stroke"[Mesh]) OR (((((((((((((((((((((((((Ischemic Strokes) OR (Stroke, Ischemic)) OR (Ischaemic Stroke)) OR (Ischaemic Strokes)) OR (Stroke, Ischaemic)) OR (Cryptogenic Ischemic Stroke)) OR (Cryptogenic Ischemic Strokes)) OR (Ischemic Stroke, Cryptogenic)) OR (Stroke, Cryptogenic Ischemic)) OR (Cryptogenic Stroke)) OR (Cryptogenic Strokes)) OR (Stroke, Cryptogenic)) OR (Cryptogenic Embolism Stroke)) OR (Cryptogenic Embolism Strokes)) OR (Embolism Stroke, Cryptogenic)) OR (Stroke, Cryptogenic Embolism)) OR (Wake-up Stroke)) OR (Stroke, Wake-up)) OR (Wake up Stroke)) OR (Wake-up Strokes)) OR (Acute Ischemic Stroke)) OR (Acute Ischemic Strokes)) OR (Ischemic Stroke, Acute)) OR (Stroke, Acute Ischemic))) OR (("Stroke"[Mesh]) OR (((((((((((((((((((((((((((((Strokes) OR (Cerebrovascular Accident)) OR (Cerebrovascular Accidents)) OR (CVA (Cerebrovascular Accident))) OR (CVAs (Cerebrovascular Accident))) OR (Cerebrovascular Apoplexy)) OR (Apoplexy, Cerebrovascular)) OR (Vascular Accident, Brain)) OR (Brain Vascular Accident)) OR (Brain Vascular Accidents)) OR (Vascular Accidents, Brain)) OR (Cerebrovascular Stroke)) OR (Cerebrovascular Strokes)) OR (Stroke, Cerebrovascular)) OR (Strokes, Cerebrovascular)) OR (Apoplexy)) OR (Cerebral Stroke)) OR (Cerebral Strokes)) OR (Stroke, Cerebral)) OR (Strokes, Cerebral)) OR (Stroke, Acute)) OR (Acute Stroke)) OR (Acute Strokes)) OR (Strokes, Acute)) OR (Cerebrovascular Accident, Acute)) OR (Acute Cerebrovascular Accident)) OR (Acute Cerebrovascular Accidents)) OR (Cerebrovascular Accidents, Acute))))
Key terms for search of Cochrane Library	
#1	MeSH descriptor: [Ischemic Stroke] explode all trees
#2	(Ischaemic Strokes):ti,ab,kw OR (Stroke, Ischaemic):ti,ab,kw OR (Cryptogenic Ischemic Stroke):ti,ab,kw OR (Cryptogenic Ischemic Strokes):ti,ab,kw OR (Ischemic Stroke, Cryptogenic):ti,ab,kw OR (Stroke, Cryptogenic Ischemic):ti,ab,kw OR (Cryptogenic Stroke):ti,ab,kw OR (Cryptogenic Strokes):ti,ab,kw OR (Stroke, Cryptogenic):ti,ab,kw OR (Cryptogenic Embolism Stroke):ti,ab,kw OR (Cryptogenic Embolism Strokes):ti,ab,kw OR (Embolism Stroke, Cryptogenic):ti,ab,kw OR (Stroke, Cryptogenic Embolism):ti,ab,kw OR (Wake-up Stroke):ti,ab,kw OR (Stroke, Wake-up):ti,ab,kw OR (Wake up Stroke):ti,ab,kw OR (Wake-up Strokes):ti,ab,kw OR (Acute Ischemic Stroke):ti,ab,kw OR (Acute Ischemic Strokes):ti,ab,kw OR (Ischemic Stroke, Acute):ti,ab,kw OR (Stroke, Acute Ischemic):ti,ab,kw
#	#1 OR #2

3	
#4	MeSH descriptor: [Stroke] explode all trees
#5	(Strokes):ti,ab,kw OR (Cerebrovascular Accident):ti,ab,kw OR (Cerebrovascular Accidents):ti,ab,kw OR (CVA (Cerebrovascular Accident)):ti,ab,kw OR (CVAs (Cerebrovascular Accident)):ti,ab,kw OR (Cerebrovascular Apoplexy):ti,ab,kw OR (Apoplexy, Cerebrovascular):ti,ab,kw OR (Vascular Accident, Brain):ti,ab,kw OR (Brain Vascular Accident):ti,ab,kw OR (Brain Vascular Accidents):ti,ab,kw OR (Vascular Accidents, Brain):ti,ab,kw OR (Cerebrovascular Stroke):ti,ab,kw OR (Cerebrovascular Strokes):ti,ab,kw OR (Stroke, Cerebrovascular):ti,ab,kw OR (Strokes, Cerebrovascular):ti,ab,kw OR (Apoplexy):ti,ab,kw OR (Cerebral Stroke):ti,ab,kw OR (Cerebral Strokes):ti,ab,kw OR (Stroke, Cerebral):ti,ab,kw OR (Strokes, Cerebral):ti,ab,kw OR (Stroke, Acute):ti,ab,kw OR (Acute Stroke):ti,ab,kw OR (Acute Strokes):ti,ab,kw OR (Strokes, Acute):ti,ab,kw OR (Cerebrovascular Accident, Acute):ti,ab,kw OR (Acute Cerebrovascular Accident):ti,ab,kw OR (Acute Cerebrovascular Accidents):ti,ab,kw OR (Cerebrovascular Accidents, Acute):ti,ab,kw
#6	#4 OR #5
#7	(Trimethyloxamine):ti,ab,kw OR (trimethylammonium oxide):ti,ab,kw OR (trimethylamine N-oxide):ti,ab,kw OR (TMAO):ti,ab,kw OR (trimethylamine oxide):ti,ab,kw
#8	#3 OR #6
#9	#7 AND #8
Key terms for search of Embase	
#1	'acute ischemic stroke'/exp
#2	'ischemic stroke':ab,ti OR 'ischemic strokes':ab,ti OR 'stroke, ischemic':ab,ti OR 'ischaemic stroke':ab,ti OR 'ischaemic strokes':ab,ti OR 'stroke, ischaemic':ab,ti OR 'cryptogenic ischemic stroke':ab,ti OR 'cryptogenic ischemic strokes':ab,ti OR 'ischemic stroke, cryptogenic':ab,ti OR 'stroke, cryptogenic ischemic':ab,ti OR 'cryptogenic stroke':ab,ti OR 'cryptogenic strokes':ab,ti OR 'stroke, cryptogenic':ab,ti OR 'cryptogenic embolism stroke':ab,ti OR 'cryptogenic embolism strokes':ab,ti OR 'embolism stroke, cryptogenic':ab,ti OR 'stroke, cryptogenic embolism':ab,ti OR 'wake-up stroke':ab,ti OR 'stroke, wake-up':ab,ti OR 'wake up stroke':ab,ti OR 'wake-up strokes':ab,ti OR 'acute ischemic stroke':ab,ti OR 'acute ischemic strokes':ab,ti OR 'ischemic stroke, acute':ab,ti OR 'stroke, acute ischemic':ab,ti
#3	#1 OR #2

# 4	'cerebrovascular accident'/exp
# 5	'stroke':ab,ti OR 'strokes':ab,ti OR 'cerebrovascular accident':ab,ti OR 'cerebrovascular accidents':ab,ti OR 'cva (cerebrovascular accident)':ab,ti OR 'cvas (cerebrovascular accident)':ab,ti OR 'cerebrovascular apoplexy':ab,ti OR 'apoplexy, cerebrovascular':ab,ti OR 'vascular accident, brain':ab,ti OR 'brain vascular accident':ab,ti OR 'brain vascular accidents':ab,ti OR 'vascular accidents, brain':ab,ti OR 'cerebrovascular stroke':ab,ti OR 'cerebrovascular strokes':ab,ti OR 'stroke, cerebrovascular':ab,ti OR 'strokes, cerebrovascular':ab,ti OR 'apoplexy':ab,ti OR 'cerebral stroke':ab,ti OR 'cerebral strokes':ab,ti OR 'stroke, cerebral':ab,ti OR 'strokes, cerebral':ab,ti OR 'stroke, acute':ab,ti OR 'acute stroke':ab,ti OR 'acute strokes':ab,ti OR 'strokes, acute':ab,ti OR 'cerebrovascular accident, acute':ab,ti OR 'acute cerebrovascular accident':ab,ti OR 'acute cerebrovascular accidents':ab,ti OR 'cerebrovascular accidents, acute':ab,ti
# 6	#4 OR #5
# 7	#3 OR #6
# 8	'trimethyloxamine':ab,ti OR 'trimethylammonium oxide':ab,ti OR 'trimethylamine n-oxide':ab,ti OR 'tmao':ab,ti OR 'trimethylamine oxide':ab,ti
# 9	#7 AND #8
Key terms for search of CNKI	
# 1	(Subject: Stroke (accurate) OR (subject: Ischemic stroke (accurate)) OR (subject: cerebral infarction (accurate)) OR (Subject: acute ischemic stroke (accurate)) AND (subject: TMAO (accurate)) OR (subject: TMAO (accurate))
Key terms for search of VIP	
# 1	Title or keyword = TMAO+trimethylamine oxide + trimethylamine oxide AND title or keyword = ischemic stroke + stroke + acute ischemic stroke + cerebral infarction
Key terms for search of CBM	
# 1	("Ischemic stroke" [Common field: intelligence] OR "Stroke" [Common field: intelligence] OR "Acute ischemic stroke" [Common field: intelligence] OR "Cerebral infarction" [Common field: intelligence]) AND ("TMAO" [common field: intelligence] OR "trimethylamine oxide" [common field: intelligence] OR "trimethylamine oxide" [common field: intelligence])
Key terms for search of Wanfang	
# 1	Subject: (TMAO OR Trimethylamine oxide) AND Subject: (Ischemic stroke OR stroke OR acute Ischemic stroke OR cerebral infarction)
