# Supplementary material for: The relationship between trimethylamine-N-oxide and the risk of acute ischemic stroke: A dose‒response meta-analysis
Source: PLoS One. 2023 Oct 26;18(10):e0293275. doi: 10.1371/journal.pone.0293275 (PMC10602245; doi:10.1371/journal.pone.0293275)
Supplement: S3 Table — (PDF) [file pone.0293275.s003.pdf]

Table 1 Basic features of the involved literature for meta-analysis.

Authors and years	Country	Type of study	Sample (AIS/control)	Sample type	Method*	Sex (male/female)		Age (years)		Means of AIS group	SDs of AIS group	Means of Control	SDs of Controls	Scores of NOS
						AIS	control	AIS	control					
Xiaohui Zhao 2021	China	Case–controls	124/118	Plasma	HPLC-MS/MS	/	/	56.9±3.2	57.4±3.5	13.97	1.26	7.95	3.18	6
Yunyun Xu 2022	China	Case–controls	108/59	Plasma	LC–MS	66/42	23/36	66.42±3.16	62.57±1.68	2763	408.7	1642	144.1	6
Yanfei Xu 2017	China	Case–controls	60/30	Plasma	HPLC-MS/MS	45/15	023/7	59(19.75)	57(15)	202.09	144.21	140.88	48.12	7
Kaicheng Wang 2020	China	Case–controls	108/59	Plasma	LC–MS	/	/	66.42±3.16	62.57±1.68	3205.51	716.39	1663	117.8	6
Jiaxin Luo 2022	China	Case–controls	137/121	Plasma	ELISA	72/65	35/86	57.9±8.0	55.4±10.00	3.21	0.33	2.86	0.31	7
Zhendong Liu 2017	China	Case–controls	80/40	Plasma	LC–MS	48/32	27/13	62.5±8.4	61.4±8.6	14.86	4.11	6.12	3.84	6
Shuoxi Liao 2016	China	Case–controls	322/231	Plasma	LC–MS	220/102	130/101	61(19)	56(11)	2.7	2.57	1.9	1.47	6
Qijin Zhai 2020	China	Case–controls	408/102	Serum	LC–MS	233/175	55/47	67.9±9.1	66.3±8.8	3.6949	1.8599	3.0056	1.7296	6
Chen Zhu 2019	China	Case–controls	256/100	Plasma	LC–MS	139/117	/	67.1±11.0	/	5.6	2.4	4.9	1.8	8

Jianli Zhang 2021	China	Cohort study	351/150	Plasma	LC/MS	177/174	75/75	66(57-74)	66(57-74)	6.5908	4.6152	4.1054	2.6199	6
Dongjuan Xu 2021	China	Case–controls	50/50	Plasma	LC–MS	35/15	14/36	63.2±12.0	57.36±10.65	2659.51	976.81	1484.75	648.87	7
Chuanjie Wu 2020	China	Case–controls	377/50	Plasma	LC–MS	215/162	/	62.5±10.7	/	5.3454	3.2	3.2477	1.7556	8
Taoping Sun 2021	China	Case–controls	953/953	Plasma	HPLC-MS/MS	544/409	544/409	63.14(9.26)	63.32(8.47)	3.0706	2.1159	2.5751	1.7076	8
Schneider C 2020 C. Schneider 2020	USA	Cohort study	193/100	Plasma	LC–MS	122/71	53/47	69(60-78)	65(57-75)	4.5042	2.7039	3.4838	2.3168	7
Maimaiti Rexidamu 2019	China	Case–controls	255/255	Serum	HPLC–MS/MS	136/119	/	65(57-71)	/	6.3963	4.9952	4.3209	2.8331	8
Zhaoguang Liang 2018	China	Case–controls	68/111	Plasma	HPLC-MS/MS	40/28	64/47	68.0±9.6	64.1±13.3	8.25	1.58	2.22	0.09	7
Zaiwang Li 2022	China	Case–controls	108/60	Plasma	HPLC-MS/MS	/	/	/	/	3.6527	3.1934	1.9007	1.4888	7
Yan-Yan Chen 2022	China	Case–controls	291/235	Plasma	LC–MS	132/159	98/137	61.68±7.27	59.71±7.67	129.65	46.24	85.15	32.11	7

Dong Liu 2022	China	Cohort study	412/412	Plasma	HPLC-MS/MS	189/223	189/223	69.3(62.7,75.3)	69.3(62.9,75.2)	3.6669	2.3888	3.0152	1.6515	7
Jing Nie 2018	China	Case–controls	622/622	Serum	LC–MS	292/330	292/330	62.2（7.3）	62.2（7.3）	/	/	/	/	8

* HPLC-MS/MS is high performance liquid chromatography-mass spectrometry or mass spectrometry. LC-MS is liquid chromatography-mass spectrometry, and ELISA means enzyme linked immunosorbent assay. AIS, acute ischemic stroke; SDs, Standard deviations; NOS, New Ottawa Scale.

Table 2 Basic features of the involved literature for dose–response meta-analysis.

Study	Country	Study type	Sample (AIS/Control)	Sex (male/female)	TMAO comparison (μmol/L)	OR (95% CI)	Adjusted variables
Taoping Sun 2021	China	Case–control	953/953	1088/818	Quintile 4 vs. 1 (<1.53 v >3.83)	1.81 (1.27, 2.59)	Adjusted for age, sex, BMI, smoking status, alcohol habit, history of hypertension, history of diabetes, triglycerides, LDL-cholesterol, and HDL-cholesterol.
Dong Liu 2022	China	Case–control	412/412	378/446	Quintile 4 vs. 1(<1.97 v >4.19)	1.74 (1.16, 2.61)	Adjusted for body mass index (continuous), smoking (yes or no), hypertension (yes or no), educational attainment (0 year, 1–5 years, or ≥6 years), and estimated glomerular filtration rate (quartiles).
Jing Nie 2018	China	Case–control	502/506	NA.	Quintile 3 vs. 1(<1.79 v ≥1.79)	1.35 (1.00, 1.81)	Adjusted for SBP, BMI, fasting glucose, total cholesterol, eGFR, total homocysteine, folate, smoking status at baseline, time-averaged SBP during the treatment period, choline and L-carnitine

AIS, acute ischemic stroke; BMI, body mass index; CI, confidence interval; eGFR, estimated glomerular filtration rate; HDL, high-density lipoprotein; LDL, low- density lipoprotein; NA, not available; OR, odds ratio; SBP, systolic blood pressure; TMAO, trimethylamine-N-oxide.
